# Contusions and Sprains of the Back, with Special Reference to the Early Treatment of These Injuries

**Published:** 1895-04

**Authors:** Henry R. Wharton

**Affiliations:** Philadelphia; Surgeon to the Presbyterian, Methodist Episcopal, and Children’s Hospitals; Demonstrator of Surgery at the University of Pennsylvania


### CONTUSIONS AND SPRAINS OF THE BACK, WITH SPECIAL REFERENCE TO THE EARLY TREATMENT OF THESE INJURIES.*

BY HENRY R. WHARTON, M.D., Philadelphia.

Surgeon to the Presbyterian, Methodist Episcopal, and Children's Hospitals; Demonstrator of Surgery at the University of Pennsylvania.

During my term of service in the Presbyterian Hospital in 1892 there were admitted to the surgical wards nine patients who suffered from contusions and sprains of the back, and it has occurred to me that a short description of the method of treatment, which I have employed with the most satisfactory results in this class of injuries, might be of some interest to the Fellows of the Academy.

CASE I.—J. P., aged twenty-eight, gardener, who was admitted to the hospital May 5, 1892, received a blow upon the back in the left lumbar region from a heavy wooden tub, which caused him severe pain. An examination after admission proved that there was no injury to the spine, but there was intense pain upon pressure in the left lumbar region, and also severe pain upon motion. The patient's back was strapped with adhesive plaster, and two

*Read before the Philadelphia Academy of Surgery.

days afterward he was able to sit up with comfort, and he was discharged from the hospital on May 9th.

CASE II.—H. P., aged nineteen years, fireman, was admitted May 12, 1892, with the following history: While standing on the edge of the tender of the locomotive he slipped and fell between the tender and the station platform; the engine was moving at the time, and he was rolled between the tender and platform, being severely squeezed in the lumbar region.

Upon examination after admission, no fracture of the spine or pelvis could be detected, but the patient complained of intense pain in the back, was unable to stand, and suffered retention of urine. The back was firmly strapped with adhesive plaster. Upon introducing a catheter, a large quantity of bloody urine was drawn from the bladder, and after this the patient passed the urine voluntarily, which was deeply tinged with blood for four days. The patient improved steadily, and was discharged from the hospital on May 27th, being able to walk, but still having some tenderness in the lumbar region, this part being still supported by means of adhesive straps.

CASE III.—J. G., aged twenty-five years, steam fitter, was admitted on May 20, 1892. The patient stated that while boarding a moving train at Powelton avenue he was thrown against the milk platform, striking his back and shoulder. An examination, on admission, showed slight contusion of the shoulder and marked contusion and tenderness over the lumbar region. The patient was unable to walk, and complained of severe pain upon pressure, and upon making any movements. The back was strapped, which gave great relief. The patient also suffered from retention of urine, requiring the use of a catheter. The patient was discharged in good condition on June 4th.

CASE IV.—D. D., aged forty-three years, bricklayer, was admitted to the hospital on May 23, 1892. The patient stated that while standing on a platform sixteen feet high, laying brick, the wall was pushed over by a derrick and he was thrown to the ground, striking upon his back. An examination after admission showed marked contusion of back and shoulder, great tenderness upon pressure and motion, and some tenderness over the spinous process of one of the lower dorsal vertebræ. The back was strapped, which gave great comfort. The patient was kept in bed for a week, and was discharged on June 6th, in good condition.

Case V.—R. C. W., aged twenty-seven years, brakeman, was admitted to the hospital on May 28, 1892. The patient stated that he was knocked off the top of a car on which he was riding, and was thrown to the ground, striking upon his back. On examination there was marked contusion of the back in the lumbar region, tenderness upon pressure, and inability to stand or walk. The patient's back was strapped, which gave him marked relief, and he was discharged on June 30th.

Case VI.—A. W., aged twenty-eight years, trucker, was admitted to the hospital on May 21, 1892. The patient stated that while moving a heavy slab or stone, it fell and struck him upon his back. An examination after admission to the hospital detected no fracture of the vertebræ, but there was soreness and tenderness on pressure in the lumbar region. The back was strapped, and the patient was discharged from the hospital on June 3d, in good condition.

Case VII.—J. C., aged nineteen years, iceman, was admitted to the hospital on June 12, 1892. The patient stated that he slipped while crossing the street, and fell, striking his back upon the curbstone. He was unable to walk, and was brought to the hospital by the patrol. Upon examination, after admission, it was found that the patient had great pain in the left lumbar region, but there was no evidence of fracture of the vertebræ. The back was strapped, which gave him immediate relief. This patient suffered from retention of urine, and upon evacuation of the bladder it was found that the urine was bloody. The blood disappeared from the urine in a few days. The patient did well, and was discharged on June 14th.

Case VIII.—W. McN., aged thirty-five years, brakeman, was admitted June 22, 1892. The patient stated that in a freight wreck at Tacony, the car on which he stood was thrown from the track, and he was thrown to the ground, striking upon his back. An examination after admission showed that he was suffering from contusion of the back and foot. The back was strapped, and the patient was discharged, in good condition, on June 25th.

CASE IX.—L. B., aged twenty-five years, was admitted to the hospital June 25, 1892. The patient stated that while standing on the top of a freight car he was knocked off by the Spring Garden street bridge, and was thrown to the ground, striking upon his back. On examination, after admission, he was found to be suffering from severe contusion of the back, but there was no evidence of fracture of the vertebræ. The back was strapped, which gave him marked relief. The patient also passed bloody urine. The patient did well, and was discharged on June 28th.

It will be noticed in the above cases that the lumbar-dorsal region of the back was the part most frequently injured, and this part seems to be that which was most commonly the seat of contusions and sprains. As regards the treatment of contusions and sprains of the back, I consider that rest in bed is a matter of the first importance, and in addition I have found that the pain and general discomfort of the patient is much diminished, and the time of treatment much shortened by having the back firmly strapped as soon as the patient comes under observation. The strapping of the back is effected by taking strips of resin-adhesive or of rubber-adhesive plaster, two and a half inches in width, and long enough to extend half way around the body; these are applied so as to cover in the back, one strap slightly overlapping the other, from a point just below the junction of the last lumbar vertebræ with the sacrum to the lower ribs. These straps were often removed at the end of two or three days, and the back was restrapped if the pain and tenderness still persisted. The straps were usually allowed to remain in place until the patient was up and about, without complaining of pain or discomfort in the region of the injury. In cases of severe contusion the straps often require renewal a number of times.

This method of treatment of contusions of the back was first called to my notice by Professor Ashhurst while serving as resident physician in his wards at the University Hospital, and since I have employed it I have entirely discarded the use of fomentations and stimulating lotions, which are generally recommended in the treatment of these injuries.

The treatment usually recommended in contusions and sprains of the back is warmth, friction, stimulating liniments, anodynes, acupuncture, galvanism, and massage, and of these I think massage is the most valuable, employed after the acute symptoms following the injury have subsided; but in early stages of these injuries I am convinced that strapping will be found the most satisfactory method of treatment.

I have observed that the application of straps employed as above described is usually promptly followed by relief of pain, and the fixation produced allows the patient to move with more comfort, and I am very certain, after now having employed this method of treatment in a considerable number of cases, that the time required for the recovery of the injured parts is much shortened. It will be observed, by referring to the cases reported, that many of them were comparatively trivial injuries, and the patients recovered in a short time; but even in this class of cases the suffering is often very intense for the first few days. It will also be observed that Cases II, VII, and IX passed bloody urine for a few days after the injury, showing that the injury had been severe enough to produce laceration or contusion of the kidney. Liddell,* in his very excellent article upon contusions and sprains of the back, speaks of the frequency of hematuria in these injuries when powerful blows have been delivered upon the lumbar or dorsal region of the back. The recovery, as far as I know, in all of the cases reported was satisfactory, except in Case IV. In this case the patient developed, some months after leaving the hospital, symptoms of traumatic neurasthenia, complaining of pain in the back and head, and vertigo, and brought suit against the contractor for whom he was working at the time of the injury. From what I heard of this case, and from the fact that when it was ascertained that the patient was doing his ordinary work the suit was settled for a trivial sum, I am inclined to think that the symptoms developed were not severe, and might be classed as litigation symptoms.

In cases of severe contusion or sprain of the back, when there is inability to stand or there is present great pain on motion, and where tenderness over the spine and a certain amount of fixation is present after the injury, I think there is too much tendency to attribute the symptoms resulting to an injury of the spinal cord or

* International Encyclopedia of Surgery, vol. iv., p. 700.

membrane, which injuries, when unaccompanied with fractures of the vertebræ, are extremely rare, whereas the injury resulting to the muscles, ligamentous structures, and nerves, with perhaps the wrenching and laceration of the vertebral articulations, is perfectly possible to account for the symptoms resulting, and I agree with Mr. Page that many of these cases are well described by the term "traumatic lumbago."

As contusions and sprains of the back are injuries which are often followed by the development of symptoms which are described as traumatic neurosis, or traumatic neurasthenia, it seems to me that these are cases which should be carefully treated when they first come under the observation of the surgeon, for I am sure that many of these cases, if so treated by rest and fixation for a short time, would make more complete recoveries, and would be less likely to develop the symptoms above described. In cases of contusions or sprains of the back in which symptoms of traumatic neurasthenia develop, and which give rise to litigation, it is often difficult for the surgeon to estimate how far the original shock of the system following the injury is responsible for the symptoms presented. In many cases the objective signs presented leave no doubt of the severe nature of the injury, while in other cases the symptoms complained of are mainly subjective in their character, and these are the cases which give rise to the most troublesome litigation. It is often difficult to decide whether the symptoms presented are merely assumed or exaggerated for fraudulent purposes, or whether, without any attempt to deception on the part of the patient, injuries trivial in themselves may be unconsciously exaggerated, and be apparently productive of serious results. Although many severe injuries of the back apparently recover without developing such symptoms as have been described, there is no doubt that the element of compensation for the suffering and disability from the injuries received plays an important part in the exaggeration of these symptoms, and that expectancy may be justly credited with an important place in their exaggeration. In cases of serious disorder resulting from contusions and sprains of the back, often apparently trivial, the symptoms developing are usually progressive in their character, and soon there will come manifestly marked objective signs, such as paralysis, disturbances of the reflexes, loss of electrical excitability, disturbances of the bladder, loss of flesh, sleeplessness, etc., which place the existence of morbid changes beyond a doubt.

